# miR-429-3p/*LPIN1* Axis Promotes Chicken Abdominal Fat Deposition via PPARγ Pathway

**DOI:** 10.3389/fcell.2020.595637

**Published:** 2020-12-21

**Authors:** Xiaohuan Chao, Lijin Guo, Qi Wang, Weiling Huang, Manqing Liu, Kang Luan, Jinqi Jiang, Shudai Lin, Qinghua Nie, Wen Luo, Xiquan Zhang, Qingbin Luo

**Affiliations:** ^1^Guangdong Laboratory for Lingnan Modern Agricultural Science and Technology, South China Agricultural University, Guangzhou, China; ^2^Guangdong Provincial Key Lab of Agro-Animal Genomics and Molecular Breeding, and Key Laboratory of Chicken Genetics, Breeding and Reproduction, Ministry of Agriculture, Guangzhou, China; ^3^College of Animal Science, South China Agricultural University, Guangzhou, China

**Keywords:** RNA-Seq, miR-429-3p/*LPIN1* axis, preadipocyte proliferation, preadipocyte differentiation, PPARγ pathway abstract

## Abstract

To explore the regulatory mechanism of abdominal fat deposition in broilers, 100-day-old Sanhuang chickens (*n* = 12) were divided into high-fat and low-fat groups, according to the abdominal fat ratio size. Total RNA isolated from the 12 abdominal fat tissues was used for miRNA and mRNA sequencing. Results of miRNA and mRNA sequencing revealed that miR-429-3p was highly expressed in high-fat chicken whereas *LPIN1* expression was downregulated. Further, we determined that miR-429-3p promoted preadipocyte proliferation and differentiation, whereas *LPIN1* exerted an opposite effect. Notably, we found that the miR-429-3p/*LPIN1* axis facilitated PPARγ pathway activation, which is closely associated with the progression of adipogenesis. In conclusion, our results provide evidence that a novel miR-429-3p/*LPIN1* axis is involved in the regulation of adipogenesis, which may have a guiding role in the improvement of breeding for abdominal fat traits in broiler chickens.

## Introduction

Obesity is becoming a world-wide health problem, which is believed to increase the risk of psychological problems, fatty liver disease, cardiovascular disease, and even cancer (Bray, 2004; Swinburn et al., 2011; Hruby and Hu, 2015; Lauby-Secretan et al., 2016; Bugianesi, 2020). The rising prevalence of this disorder is arousing the concern and awareness of the public, as well as piquing the attention and interest of researchers (James, 2018; Jaacks et al., 2019; Saxton et al., 2019). The wide availability of affordable and high-fat foods and beverages are contributing to excess energy intake and increased body weight (Liu et al., 2016; Asghari et al., 2017; Heianza and Qi, 2017) with sedentary lifestyles contributing to the weight issue (Barnett et al., 2018; Lavie et al., 2019). In addition to an unhealthy lifestyle, obesity is believed to be highly associated with genetic manipulation (Goodarzi, 2018; Qasim et al., 2018). In vertebrates, adipogenesis is a complex process involving the regulation of multiple gene expression networks and various metabolic pathways (Homan et al., 2011; Bugianesi, 2020). A number of obesity-associated genes have been identified in adipogenesis regulation, such as melanocortin-4 receptor (MC4R) (Kuhnen et al., 2019), uncoupling protein-1 (UCP1) (Fedorenko et al., 2012), and pro-opiomelanocortin (POMC) (Qiu et al., 2014). Additionally, peroxisome proliferator-activated receptor gamma (PPARγ) (Brun et al., 1996) and CCAAT/enhancer-binding protein alpha (CEBPα) (Lefterova et al., 2008) have been shown to possess the ability to mediate the terminal differentiation of adipose tissue/cell development. In the poultry industry, excessive deposition of fat in commercial broilers can lead to low economic efficiency since the public tend to favor low-fat content chicken (Fouad and El-Senousey, 2014). A diet of high-fat chicken would encourage excess weight gain, posing a risk to people’s health. Therefore, investigation into obesity utilizing a chicken model can gain new insights into the mechanism underlying fat deposition and provide a possible solution for the public obesity problem.

In the past decade, the ability of microRNAs (miRNAs) and small non-coding RNAs to exert their biological functions by binding to 3’-untranslated regions (3’-UTRs) of target mRNAs, causing translational repression or degradation has become widely accepted (Bartel, 2004). Despite a lack of protein-coding ability, miRNAs are reported to play irreplaceable roles in various pathological and physiological processes including adipogenesis, metabolism and tumorigenesis (Krol et al., 2010; Li et al., 2012; Mohr and Mott, 2015; Subramaniam et al., 2019). Using miRNA sequencing analyses, we determined miR-429-3p to be a miRNA of interest for exploring the regulation of adipogenesis in chicken. miR-429 is a member of the highly conserved miR-200 family (Hasuwa et al., 2013). To date, most of the research on miR-429-3p has focused on cancer development, whereas little is known about its role in adipogenesis. The present study is the first to shed light on the effect of miR-429-3p in the mediation of adipogenesis.

*LPIN1* encodes lipin-1 protein, which is reported to be responsible for lipidic and metabolic homeostasis (Phan et al., 2004). We observed a downregulation in *LPIN1* in the abdominal fat tissue from high-fat chicken. In addition, *LPIN1* is predicted to be a potential target of miR-429-3p in the regulation of adipogenesis in chicken.

It is well-established that PPARγ is a predominant regulator of adipocyte differentiation (Tontonoz et al., 1994). In the process of adipogenesis, PPARγ works in synergy with CEBPα (which is also a well- known key regulator of adipocyte differentiation) to activate many other relevant molecular factors and contribute to lipid accumulation (Madsen et al., 2014). Abnormal expression of PPARγ in humans and insufficiency of PPARγ in mice can result in lipodystrophy (Campeau et al., 2012), indicating sufficient PPARγ expression is indispensable to the maintenance of adipogenesis.

To our best knowledge, there is no published study elucidating how the miR-429-3p/*LPIN1* axis interacts with the PPARγ pathway in the adipogenesis of chicken. Based on sequencing data, we aimed to identify the potential role of miR-429-3p and *LPIN1* in adipogenesis in a chicken model, which may better our understanding of the genetic regulatory networks in adipogenesis.

## Materials and Methods

### Animal Experiment and Ethics Statement

The animals used in this study were supported by Wens. Twelve Sanhuang broilers were put in a cage after birth and were fed *ad libitum*. When they reach the age of 100 days, they were euthanized by cervical dislocation. Body weight and abdominal fat weight were weighed. The abdominal fat tissues were collected and saved in a −80°C refrigerator. The body weight and abdominal fat weight of 12 individuals were listed in Table 1. The animal experiments conducted in this study were approved (license ID: SCAU#2017015; September 13, 2017) by the Animal Care Committee of South China Agricultural University.

**TABLE 1:** Abdominal fat rate of 12 Sanhuang broilers.

Individual ID	Weight (g)	Abdominal fat weight (g)	Abdominal fat rate (%)
L1	1705.9	66.4	3.89
L2	1550.0	56.8	3.66
L3	1360.0	50.3	3.70
L4	1564.8	58.2	3.72
L5	1419.9	55.6	3.92
L6	1235.2	34.7	2.81
H1	1602.5	128.8	8.04
H2	1747.2	146.5	8.38
H3	1415.8	119.9	8.47
H4	1735.5	151.6	8.74
H5	1618.9	155.5	9.61
H6	1839.7	179.4	9.75

### Whole Genome miRNA and mRNA Screen

Twelve 100-day-old *ad libitum* Sanhuang broilers were euthanized and abdominal fat tissue was collected. The animals were divided into the high-fat group and the low-fat group based on the abdominal fat ratio. Total RNA was extracted from the 12 abdominal fat tissues, were sent to BGI Technology for miRNA and mRNA sequencing.

### Cell Culture and Cell Transfection

Immortalized chicken precursor adipocytes (ICP-1) was provided by Hui Li research group from Northeast Agricultural University (Heilongjiang, China). ICP-1 was cultured in DMEM/F12 medium (Gibco, CA, United States) with 15% fetal serum (Gibco, CA, United States) and 1% streptomycin/penicillin (Invitrogen, CA, United States) at 37°C with 5% CO_2_. The DMEM (Gibco, CA, United States) with 10% fetal serum and 1% streptomycin/penicillin was used for culturing dermal fibroblast (DF-1) cells. The culture conditions for DF-1 were the same as for ICP-1. Lipofectamine 3000 kit (Invitrogen, CA, United States) was used for cell transfection of plasmids and oligonucleotides described in next section as per the manufacturer’s protocol. Transfection dose of the plasmids or oligonucleotides for different plates was as follows: 2.5 μg/well or 125 nM/well for 6-well plates, 1 μg/well or 50 nM/well for 12-well plates, and 0.25 μg/well or 12.5 nM/well for 48-well plates.

### Plasmids Construction and Oligonucleotides Synthesis

The complete CDS sequence of *LPIN1* (XM_004935773.3) was synthesized by GeneCreate (Hubei, China) and subcloned into *Xba*I and *Xho*I site in pcDNA3.1 vector (Promega, WI, United States). The plasmid was named as pcDNA3.1- LPIN1. Further, gga-miR-429-3p mimic, gga-miR-429-3p inhibitor, and siRNA of *LPIN1* were synthesized by RioBio (Guangdong, China). Oligonucleotides sequences used are listed in Table 2.

**TABLE 2:** Oligonucleotides sequence information.

Fragment name	Fragment sequence (5′-3′)
gga-miR-429-3p mimic	UAAUACUGUCUGGUAAUGCCGU
gga-miR-429-3p inhibitor	ACGGCAUUACCAGACAGUAUUA
si-LPIN1	GTTCCTTGATCAAAGCTAA

### Total RNA Extraction

MagZol Reagent (Magen, Guangdong, China) was used for extracting total RNA from abdominal fat tissues and cells according to manufacturer’s instructions. A total of 50 mg homogenized tissues or 10^6^ cells were lysed by adding 1 mL MagZol Reagent at 25°C. Next, 200 μL chloroform (Damao, Tianjin, China) was added to the mixture and centrifuged at 12,000 × *g* at 4°C for 15 min. The supernatant was transferred to a new 1.5 mL tube, to which 500 μL isopropyl alcohol (Damao, Tianjin, China) was added, mixed well, and incubated at room temperature for 10 min. The mixture was centrifuged at 12,000 × *g* at 4°C for 10 min and the supernatant was discarded. Then, 1 mL of 75% alcohol (Damao, Tianjin, China) was used to wash the pellet followed by centrifugation. Finally, 40 μL RNase-free water (TsingKe, Beijing, China) was used to dissolve the RNA pellet and stored at −80°C until further analysis.

### Reverse Transcription and Real-Time Quantitative PCR (qPCR)

HiScript^®^ II Q RT SuperMix for qPCR (+ gDNA wiper) (Vazyme, Jiangsu, China) and miRNA 1st Strand cDNA Synthesis Kit (by stem-loop) (Vazyme, Nanjing, China) were used for reverse transcription PCR for mRNA and miRNA, respectively, following manufacturer’s protocol. Quantitative PCR (qPCR) was performed using ABI QuantStudio 5 instrument (Thermo Fisher, NY, United States) by using ChamQ Universal SYBR qPCR Master Mix (Vazyme, Nanjing, China). GAPDH and U6 were used as internal controls for mRNA and miRNA qPCR, respectively. The primers used in qPCR were designed by Premier Primer 5.0 software and the primer sequences are listed in Supplementary Table 1.

### Flow Cytometry Analysis

After 48 h of transfection, 0.25%-EDTA trypsin (Gibco, CA, United States) was used to digest the cells and 1 mL culture medium with 15% fetal serum was used to terminate the digestion reaction. The cell suspension was centrifuged at 2,000 × *g* at 4°C for 5 min. The cell pellet was washed twice with 1 mL ice-cold PBS (Gibco, CA, United States). Subsequently, the cells were fixed overnight with 70% alcohol (Damao, Tianjin, China). Next, 1 mL ice-cold PBS (Gibco, CA, United States) was used to wash cells sediment and cells were stained with 0.5 mL Propidium iodide (PI) solution (Beyotime, Shanghai, China) at 37°C in dark for 30 min. Finally, the cells were analyzed by a CytoFlex instrument (Beckman, CA, United States).

### Oil Red O Staining

The transfected cells were induced to differentiate into adipocytes by the culture medium with 15% fetal serum and 0.2% oleic acid (Sigma, CA, United States). The cells were washed with PBS and then, 4% formaldehyde was added to fix the cells for 30 min. Next, the cells were washed with PBS, followed by wash with 60% isopropanol, and finally stained with oil red O reagent for 30 min. The cells were then treated with 60% isopropanol for 15 s and rinsed with PBS 3 times for 3 min each time. The stained cells were observed, and images were captured under an electron microscope (Nikon, Tokyo, Japan). Oil red O dye was extracted from the cells in isopropanol solution and quantified in a microreader (Bio-Rad, CA, United States) at 510 nm. Cells were collected and resuspended in a 1.5 mL tube with 1 mL PBS. Finally, cells were counted in a cell counter (CountStar, Shanghai, China).

### 5-Ethynyl-2′-Deoxyuridine (EdU) Assay

Cell-Light EdU Apollo567 *In Vitro* Kit (RioBio, Guangdong, China) was used to detect the cell proliferation activity by following manufacturer’s protocol. After staining, cells were captured in at least three random visual fields using a DMi8 microscope (Leica, Wetzlar, Germany).

### Western Blotting

Protein was extracted from the cells inoculated in a 6-well plate that had been transfected for 48 h by using ice-cold radio immunoprecipitation (RIPA) lysis buffer (Beyotime, Shanghai, China) with 1 mM phenylmethyl sulfonyl fluoride (Beyotime, Shanghai, China). Protein samples were separated on a 12% SDS-PAGE gel (EpiZyme, Shanghai, China) run at 120 V for 60 min. Then, the proteins were transferred onto the polyvinylidene fluoride (PVDF) membrane (Bio-Rad, CA, United States). After 30 min blocking with 5% skim milk, the membrane was incubated with anti-LPIN1 (1:1,000; 863631, ZENBIO), anti-PPARγ (1:1,000; bs-0530R, BIOSS), anti-CEBPα (1:1,000; LS-B4685, LABIO) or anti-GAPDH antibodies (1:2,000; bsm-33033M, BIOSS) at 4°C overnight. Anti-mouse IgG HRP-antibody (1:10,000; 7076P2, CST) or anti-rabbit IgG HRP-antibody (1:10,000; 7074P2, CST) were used as secondary antibodies. DAB Horseradish Peroxidase Color Development Kit (Beyotime, Shanghai, China) was used for chromogenic reaction following manufacturer’s protocol. Finally, protein bands were visualized using Odyssey instrument (Li-cor, CA, United States) and the gray value (Image density) of protein bands was measured by ImageJ software.

### Dual-Luciferase Reporter Assay

The wild type LPIN1 3’ UTR (LPIN1-WT) containing miR-429-3p binding sites and mutant type of LPIN1 3’ UTR (LPIN1-MT) with altered miR-429-3p binding sites were amplified by PCR and subcloned into pmirGLO vector (Promega, WI, United States). 4 × 10^4^ ICP-1 were seeded into a 96-well plate and co-transfected with miR-429-3p mimic or mimic NC with LPIN1-WT or LPIN1-MT. Firefly and Renilla Luciferase activity was detected using Dual-GLO Luciferase Assay System kit (Promega, WI, United States) in a Fluorescence/Multi-Detection Microplate Reader (BioTek, VT, United States); firefly luciferase activity was normalized to Renilla luminescence.

### Data Analysis

The data represents mean ± SEM. Student’s *t*-test and ANOVA-analysis were performed to determine the statistical significance of differences observed between groups. ^∗^*P* < 0.05; ^∗∗^*P* < 0.01; ^∗∗∗^*P* < 0.001; ^****^P < 0.0001; ns: no significance.

## Results

### Genome-Wide mRNA Screen and Analysis Identifies Several Differentially Expressed Genes in High-Fat Tissues

**FIGURE 1:**
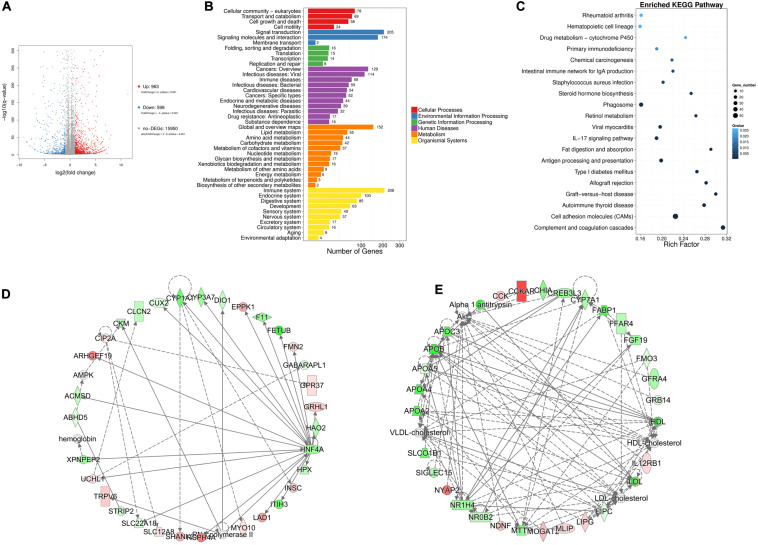
mRNA-seq analysis in chicken abdominal fat tissues between high-fat group and low-fat. **(A)** Volcano plot for low-fat_VS_high-fat DEGs. **(B)** The KEGG pathways classification analysis for DEGs. **(C)** The KEGG pathways enrichment analysis for DEGs. **(D,E)** The interaction networks for lipid metabolism-related DEGs with IPA enrichment score above 30.

To explore the regulatory mechanisms underlying chicken abdominal fat deposition, mRNA sequencing was performed. The abdominal fat tissues from 6 High fat (HF) broilers and 6 Low fat (LF) broilers were collected for mRNA sequencing to perform the genome-wide mRNA screen. Using mRNA sequencing, we obtained at least 21.94 M raw reads for each sample (Supplementary Table 2) and all raw data was submitted to the NCBI SRA database (accession number: PRJNA656618; link^1^). The clean data was mapped to chicken GRCg6a genome and gene set analysis showed that a total of 18,165 genes were detected. | Fold change | ≥ 2 and *q*-value ≤ 0.001 were considered as the cut-off for differentially expressed genes (DEGs), and we obtained 1,562 DEGs from mRNA sequencing. Compared with the LF group, 963 genes were upregulated in HF group whereas 599 genes were down-regulated (Figure 1A). We performed classification and enrichment analysis of KEGG biological pathways for these DEGs. A total of 55 and 42 genes were classified into the lipid metabolism and carbohydrate metabolism pathways, respectively (Figure 1B). The top 20 enriched KEGG pathways are shown in Figure 1C, including *complement and coagulation cascades*, *graft-versus-host disease*, and *fat digestion and absorption* among others. Furthermore, additional pathways that might be directly or mediately related to fat deposition have been listed in Supplementary Table 3. Another pathway enrichment analysis was performed using DAVID 6.8 platform, which showed that these DEGs were enriched in *steroid hormone biosynthesis*, *metabolic pathways*, *PPAR signaling*, *ECM-receptor interaction*, and *glycerolipid metabolism* pathways (Supplementary Table 4). The genes at the intersection of the two kinds of pathway enrichment analysis were selected. These DEGs related to fat formation and lipid metabolism are listed in Table 3, and were considered as potential candidates for abdominal fat deposition.

**TABLE 3:** The potential candidates for abdominal fat deposition.

Gene name	Low Fat Expression	High Fat Expression	log2Ratio (HF/LF)	*Q* value	Up Down	Gene description
ABHD5	55981	23916	–1.23016	0	Down	adenosine A1 receptor
LPIN1	48475	11944	–2.02416	0	Down	lipin 1
ACBD5	39718.81	19735.03	–1.01227	0	Down	acyl-CoA binding domain containing 5
APOC3	8831	169	–5.71069	0	Down	apolipoprotein C3
APOB	8679	40	–7.76459	0	Down	apolipoprotein B
ADORA1	8141	3018	–1.43482	0	Down	abhydrolase domain containing 5
APOA4	6931	96	–6.17709	0	Down	apolipoprotein A4
FABP1	1328	21	–5.98593	0	Down	fatty acid binding protein 1
DHCR24	1299.03	3065.5	1.235484	4E-160	Up	24-dehydricholesterol reductase
HSD11B1b	837	125	–2.7465	1.5E-128	Down	hydroxysteroid (11-beta) dehydrogenase 1b
APOA5	820	338	–1.2818	1.56E-46	Down	apolipoprotein A5
SQLE	713	1538	1.105878	3.64E-68	Up	squalene epoxidase
SLC27A2	708	320	–1.14888	3.17E-34	Down	solute carrier family 27
ACSL6	193.08	405.77	1.06826	3.51E-18	Up	long-chain-fatty acid CoA ligase 6
HNF4A	96.42	8.98	–3.42775	1.51E-19	Down	Hepatocyte nuclear factor 4 alpha
ELOVL2	55	1	–5.78456	1.74E-14	Down	ELOVL fatty acid elongase 2
CYP7A1	22	3	–2.87767	4.5E-05	Down	cytochrome P450 family 7 subfamily A member 1

In addition, ingenuity pathway analysis (IPA) was performed to investigate the gene networks for the 1,562 DEGs. In IPA, we paid more attention to the pathways involved in fat deposition, such as, lipid metabolism, insulin receptor signaling adipogenesis pathway, and fatty acid β-oxidation I (Supplementary Table 5). Three networks were involved with lipid metabolism. Here, only the two networks with the enrichment degree score above 30 are shown (Figures 1D,E). Among the DEGs, *HNF4A*, *APOB*, *NR1H4*, *LIPC*, and *LDL-cholesterol* acted as the node genes.

Finally, we compared candidate DEGs from the KEGG, DAVID, and IPA analysis platforms that associated with fat deposition. The results showed 7 common DEGs, including *SLC27A2*, *ACSL6*, *ABHD5*, *LPIN1*, *ADORA1*, *HNF4A*, and *SQLE*.

### Sequencing Analysis of miRNA Expression in High-Fat Broilers and Low-Fat Broilers

**FIGURE 2:**
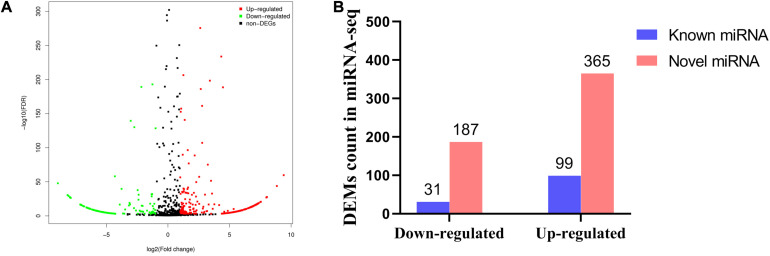
Differentially expressed miRNA in abdominal fat between low fat broilers and high fat broilers. **(A)** Volcano plot for low-fat_VS_high-fat DEMs. **(B)** The known and novel miRNAs (down-regulated or up-regulated) from DEMs.

To clarify the underlying mechanism of abdominal fat deposition from an epigenetic perspective, abdominal fat tissues from 6 HF broilers and 6 LF broilers were collected for miRNA sequencing. Small RNA sequencing raw data was submitted to the NCBI SRA database (accession number: PRJNA657369)^2^. In total, we detected 1,841 miRNAs, including 886 known miRNAs and 955 predicted miRNAs (Supplementary Table 6). The first nucleotide bias of known miRNAs showed that miRNA sequence length was approximately 21–23 nt, with 22 nt being the maximum size (Supplementary Figure 1). A total of 682 differentially expressed miRNAs (DEMs) were identified between the HF and LF group, including 130 known miRNAs. Of these, 99 known miRNAs were upregulated, while 31 known miRNAs were downregulated (Figure 2). The top 20 abundant DEMs are listed in Table 4 and included miR-126-5p, miR-148a-3p, miR-148b-3p, miR-122b-3p, miR-429-3p, miR-122-5p, miR-196-5p, and miR-1416-5p. Among upregulated miRNAs, miR-126-5p, miR-148a-3p, miR-148b-3p, and miR-429-3p were most abundant, accounting for 259,237, 180,829, 138,036, and 62,352 miRNA reads from HF group, respectively. Among the downregulated miRNAs, miR-122b-3p, miR-122-5p and miR-1416-5p were most abundant, with miR-122b-3p being the most abundant in LF group at 834,699 reads.

**TABLE 4:** The top 20 abundant DEMs in HF_VS_LF.

miRNA id	Read count (Low fat)	Read count (High fat)	log2Ratio (High fat/Low fat)	Up down regulation	*Q* value
gga-miR-122b-3p	834699	105526	–2.93401	DOWN	0
gga-miR-126-5p	132045	259237	1.022885	UP	0
gga-miR-122-5p	318046	43784	–2.81112	DOWN	0
gga-miR-148a-3p	73733	180829	1.343887	UP	0
gga-miR-148b-3p	44329	138036	1.688365	UP	0
gga-miR-1416-5p	93406	30166	–1.58095	DOWN	0
gga-miR-429-3p	20400	62352	1.66151	UP	0
gga-miR-196-5p	9988	34948	1.856585	UP	0
gga-miR-200a-3p	2842	27316	3.314411	UP	0
gga-miR-200b-3p	4731	21781	2.252496	UP	0
gga-miR-375	13756	3176	–2.06514	DOWN	0
gga-miR-130c-3p	4342	8549	1.027039	UP	0
gga-miR-135a-5p	2506	9942	2.037793	UP	0
gga-let-7l-5p	224	11707	5.757372	UP	0
gga-miR-15b-5p	3518	8147	1.261156	UP	0
gga-miR-34b-3p	1988	9386	2.288836	UP	0
gga-miR-183	7954	3256	–1.23894	DOWN	0
gga-miR-449a	841	6271	2.948161	UP	0
gga-miR-142-5p	1661	4978	1.633157	UP	0
gga-miR-122-3p	4703	1044	–2.12182	DOWN	0

### The Co-analysis Between DEMs and DEGs Revealed miR-429-3p/*LPIN1* Axis May Regulate Adipogenesis

**FIGURE 3:**
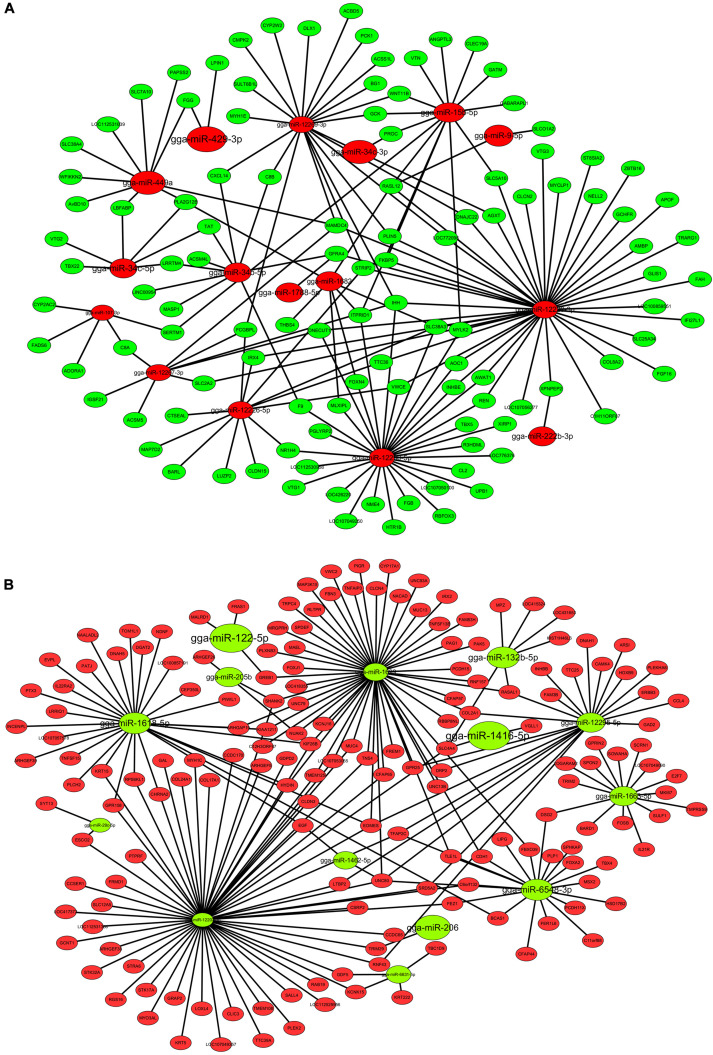
DEMs-DEGs interaction networks. **(A)** The interaction network between up-regulated DEMs and down-regulated DEGs. **(B)** The interaction network between down-regulated DEMs and up-regulated DEGs. The pattern size of DEMs was determined by miRNA abundance the pattern size of DEGs is uniform.

To further analyze the interactions between specific miRNAs and their target genes during abdominal fat deposition, we performed a DEMs-DEGs co-analysis. Considering the negative regulation of target mRNAs by miRNAs, the target prediction was conducted between upregulated DEMs and downregulated DEGs, or downregulated DEMs and upregulated DEGs. We obtained 1,113 target pairs, among which, 62 upregulated DEMs could target 229 DEGs that formed 801 negatively correlated target pairs. Similarly, 312 target pairs were formed by 25 downregulated DEMs and 222 DEGs. Based on the target relationship between DEMs and DEGs, we constructed two miRNA-gene interaction networks. As shown in Figure 3A, there are 16 up-regulated miRNAs and 118 DEGs in this network. miR-12229-3p and miR-12290-5p target multiple DEGs and may play a significant role. Further, Figure 3B shows that 14 downregulated miRNAs can interact with 176 DEGs. Thus, it can be concluded that miR-12207-3p and miR-1699 are critical nodes and target multiple common DEGs. Considering the candidate genes screened in mRNA sequencing, we inferred that miR-429-3p and miR-107-3p can target *LPIN1* and ADORA1, respectively, which are related to lipid metabolism. Compared with miR-429-3p/*LPIN1* axis, both miR-107-3p and *ADORA1* have lower abundance. Therefore, miR-429-3p/*LPIN1* axis may be an important regulator of adipogenesis, and we focused on this axis in follow-up experiments.

### miR-429-3p Facilitates Preadipocyte Proliferation

**FIGURE 4:**
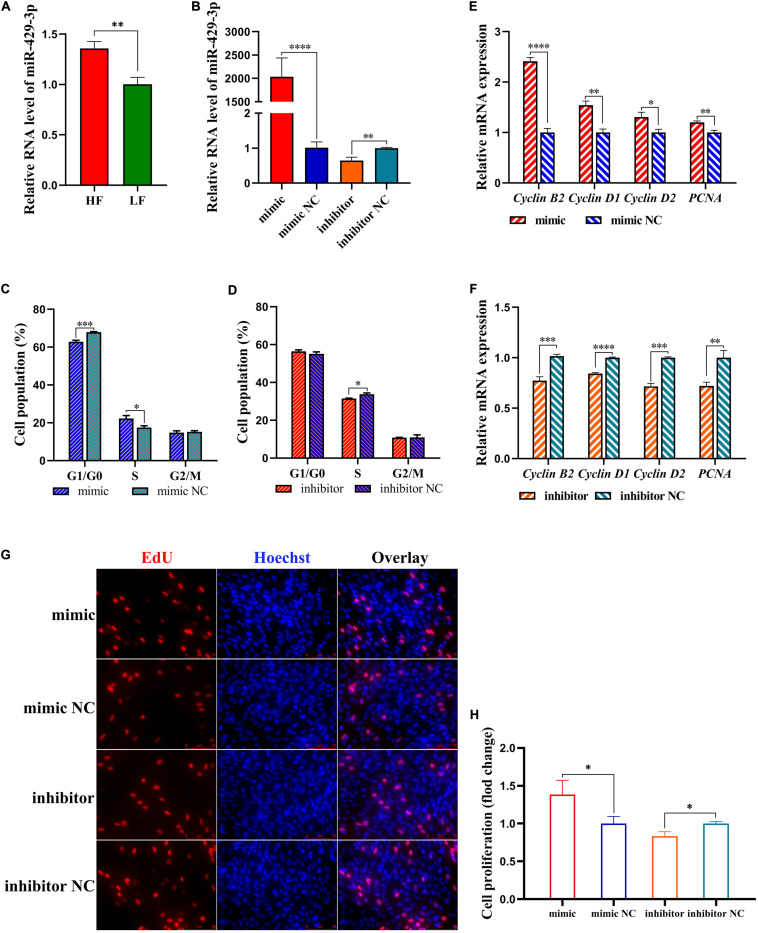
miR-429-3p promotes preadipocytes proliferation. **(A)** Relative expression of miR-429-3p in high-fat chicken and low-fat chicken. **(B)** Transfection efficiency of miR-429-3p mimic and inhibitor. **(C,D)** Flow cytometry for cell cycle detection of ICP-1 cells. **(E,F)** Relative mRNA expression of *Cyclin B2*, *Cyclin D1*, *Cyclin D2*, and *PCNA* in ICP-1 cells. **(G,H)** EdU staining for cell proliferation of ICP-1 cells. Data presented as mean ± SEM, *P < 0.05; **P < 0.01; ***P < 0.001; ****P < 0.0001; ns: no significance.

The differential expression of miR-429-3p in abdominal fat tissues of high-fat and low-fat animals was experimentally verified by qPCR (Figure 4A). A miR-429-3p mimic and miR-429-3p inhibitor were synthesized to explore the role of miR-429-3p in preadipocyte proliferation and differentiation. Our results showed that miR-429-3p mimic increased the expression of miR-429-3p in ICP-1 cells by as much as 2,000 folds compared with the control group. The miR-429-3p inhibitor could significantly knock down the endogenous expression of miR-429-3p, with an efficiency of more than 30% (Figure 4B). Flow cytometry analysis showed that miR-429-3p overexpression promoted cell cycle transition from G1 to S phase, while miR-429-3p knockdown arrested the cell cycle in the G1 phase, resulting in delayed cell cycle progression (Figures 4C,D). In addition, we also found that miR-429-3p overexpression upregulated transcription of cell cycle-promoting genes, including *cyclin B2*, *cyclin D1*, *cyclin D2*, and *PCNA* (Figures 4E,F). EdU assay performed to observe the role of miR-429-3p in preadipocyte proliferation confirmed the positive role of miR-429-3p in preadipocyte proliferation (Figures 4G,H). Thus, our results provide evidence that miR-429-3p can promote preadipocyte proliferation, a major process in abdominal fat deposition.

### miR-429-3p Contributes to Preadipocyte Differentiation via PPARγ Pathway

**FIGURE 5:**
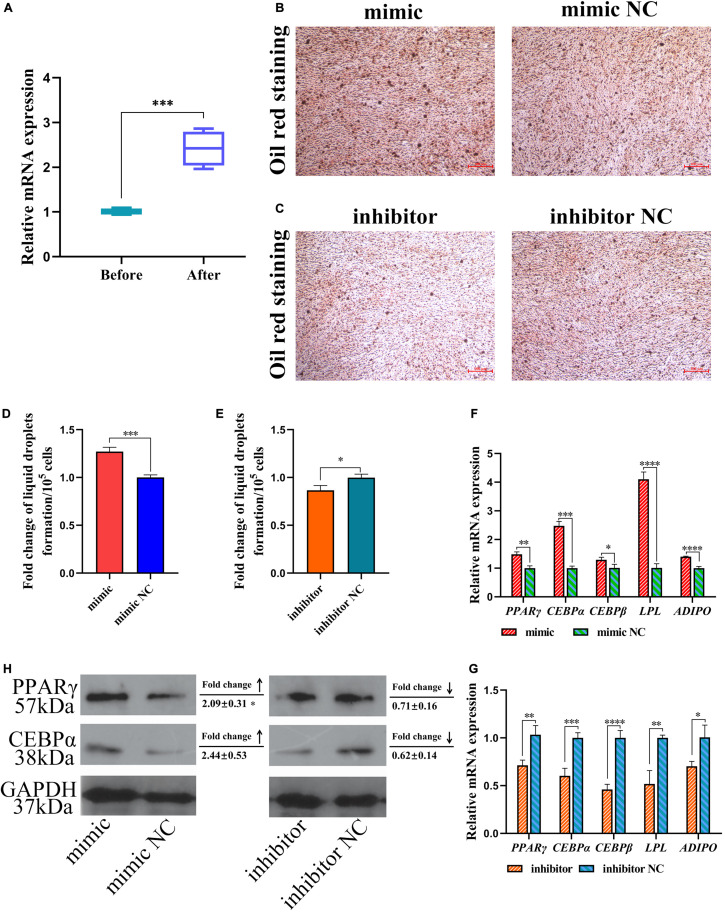
miR-429-3p promotes preadipocytes differentiation via PPARγ pathway. **(A)** Relative expression of miR-429-3p before and after preadipocytes differentiation. **(B,C)** Oil red staining for lipid droplet formation. **(D,E)** The fold change of lipid droplets formation per 10^5^ cells. **(F,G)** Relative mRNA expression of *PPAR*γ, *C/EBP*α, *C/EBP*β, *LP*L, and *ADIPO* in in ICP-1 cells. **(H)** The western blot for PPARγ and C/EBPα proteins in ICP-1 cells. Data presented as mean ± SEM, *P < 0.05; **P < 0.01; ***P < 0.001; ****P < 0.0001; ns: no significance.

The differentiation of preadipocytes to adipocytes is a critical process in abdominal fat deposition. In our study, miR-429-3p expression was detected before and after inducing preadipocyte differentiation. We observed that miR-429-3p was upregulated after preadipocyte differentiation (Figure 5A). The upregulation of miR-429-3p in preadipocyte differentiation and in the abdominal fat tissue of high-fat animals indicated that miR-429-3p was likely to play a positive role in abdominal fat deposition. We further verified whether miR-429-3p had an effect on the lipid droplet formation during preadipocyte differentiation. Compared with the control group, miR-429-3p overexpression enhanced lipid droplet formation during preadipocyte differentiation (Figures 5B,D); in contrast, the knockdown of miR-429-3p suppressed the formation of lipid droplets (Figures 5C,E). Collectively, these results demonstrate a positive effect of miR-429-3p on promoting lipid droplet formation during the adipocyte differentiation.

Next, we investigated expression of the preadipocyte differentiation-related *C/EBP*α and *C/EBP*β by qPCR. Considering that PPARγ pathway is widely regarded as a key process affecting adipocyte differentiation, we also verified the expression of genes involved in adipocyte differentiation in PPARγ pathway. After miR-429-3p mimic transfection, the mRNA levels of *C/EBP*α and *C/EBP*β were significantly upregulated (Figure 5F). On the contrary, the knockdown of miR-429-3p reduced their expression (Figure 5G). Additionally, as shown in Figure 5H, the C/EBPα and PPARγ protein levels rose substantially after miR-429-3p overexpression, while miR-429-3p knockdown reduced the protein levels of C/EBPα and PPARγ. The change in protein levels of C/EBPα and PPARγ modulated by miR-429-3p indicates that miR-429-3p promotes preadipocyte differentiation by activating PPARγ pathway.

### *LPIN1* Is a Target Gene of miR-429-3p

**FIGURE 6:**
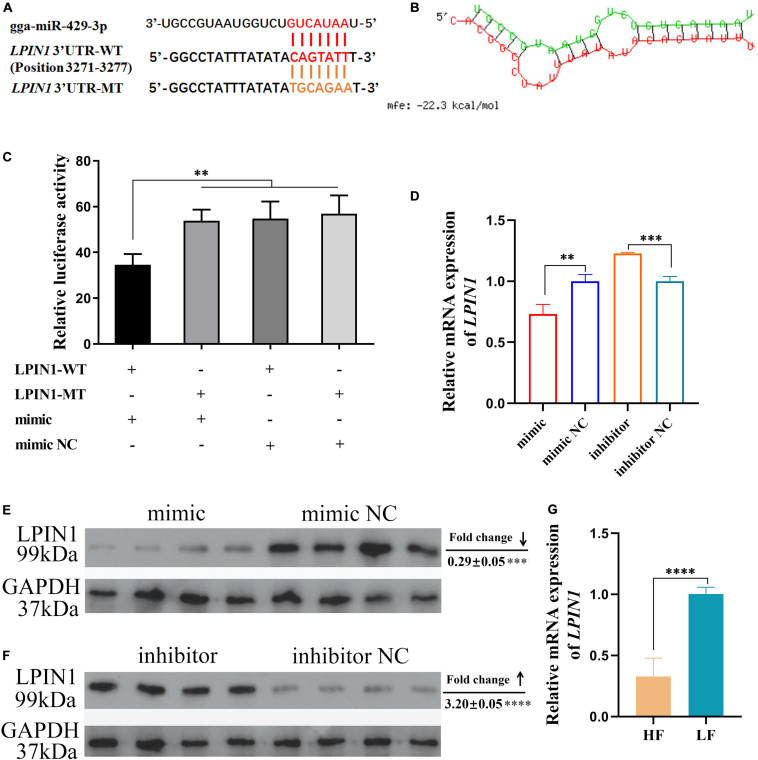
*LPIN1* is a target gene of miR-429-3p. **(A)** The target relationship prediction between miR-429-3p and *LPIN1* 3’UTR. **(B)** The stability prediction between miR-429-3p and *LPIN1* 3’UTR. **(C)** Dual-luciferase reporter assay for miR-429-3p and *LPIN1* 3’UTR. **(D)** Relative mRNA expression of *LPIN1* in ICP-1 cells treated with miR-429-3p mimic or inhibitor. **(E,F)** Relative protein level LPIN1 in ICP-1 cells treated with miR-429-3p mimic or inhibitor. **(G)** Relative mRNA expression of *LPIN1* in high-fat chicken and low-fat chicken. Data presented as mean ± SEM, *P < 0.05; **P < 0.01; ***P < 0.001; ****P < 0.0001; ns: no significance.

*LPIN1* was predicted as a target of miR-429-3p (Figure 3A). The prediction result showed that miR-429-3p can target and bind to the position 3271-3277 of *LPIN1* 3’ UTR (Figure 6A). We also performed the target pair stability analysis and the results showed that the binding between miR-429-3p and *LPIN1* 3’ UTR is stable (Figure 6B). At the cellular level, we performed a dual-luciferase reporter system analysis to verify the target relationship between miR-429-3p and *LPIN1* 3’ UTR. Compared with other groups, the co-transfection of miR-429-3p mimic and LPIN1-WT plasmid led to a lower fluorescence intensity, indicating that miR-429-3p was indeed capable of binding to *LPIN1* 3’ UTR (Figure 6C). Further, qPCR and western blot were performed to verify whether miR-429-3p overexpression or knockdown affected LPIN1 expression at mRNA and protein levels. As shown in Figure 6D, *LPIN1* expression was downregulated when miR-429-3p was overexpressed; in contrast, the expression was upregulated when miR-429-3p was knocked down. Moreover, miR-429-3p hindered the translation of *LPIN1* mRNA, leading to a decrease in the protein level of LPIN1 (Figures 6E,F). Results of RNA-seq showed that *LPIN1* was downregulated in abdominal fat tissues of high-fat animals. We also quantified the relative mRNA expression in abdominal fat tissues from the two groups by qPCR and observed lower expression of *LPIN1* in high-fat individuals (Figure 6G). Our results confirmed that *LPIN1* is a target gene of miR-429-3p and that its expression is modulated by miR-429-3p.

**FIGURE 7:**
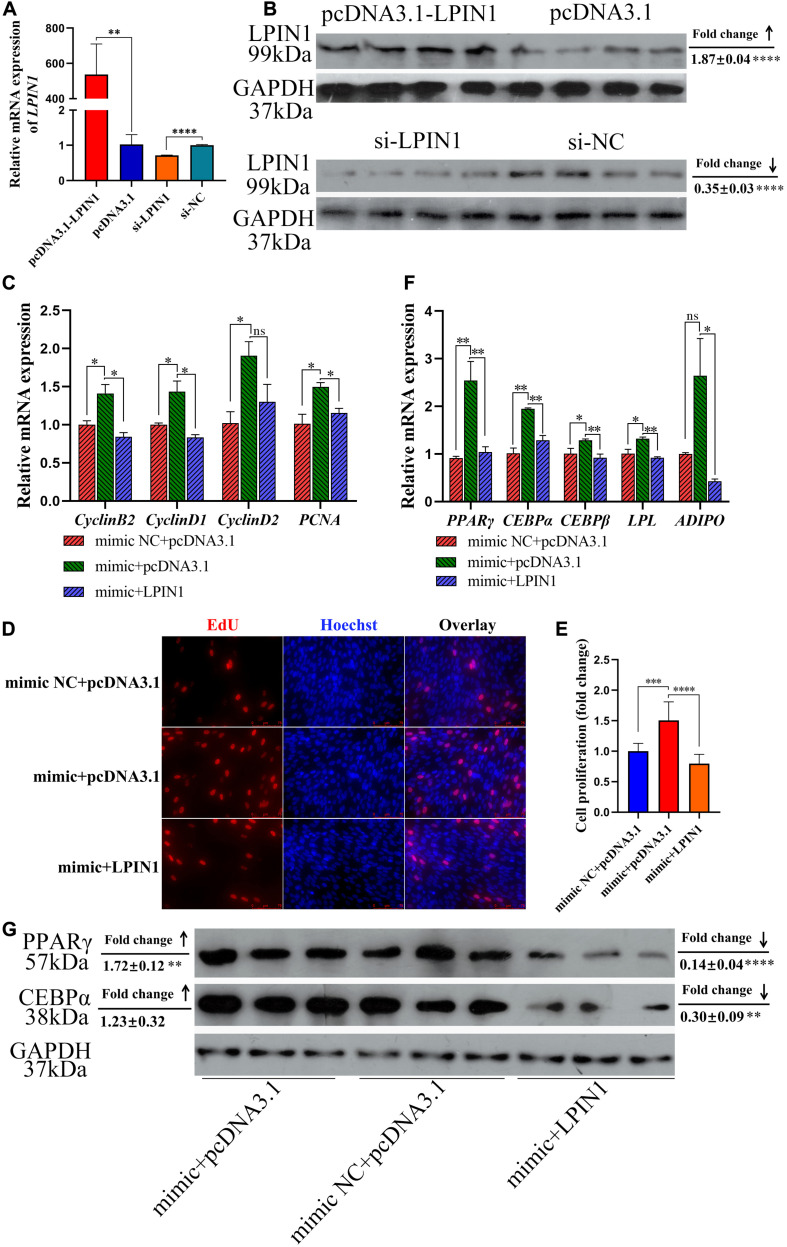
*LPIN1* plays a pivotal role on preadipocytes proliferation and differentiation regulated by miR-429-3p. **(A)** Transfection efficiency of pcDNA3.1-LPIN1 and si-LPIN1 on mRNA level. **(B)** Transfection efficiency of pcDNA3.1-LPIN1 and si-LPIN1 on protein level. **(C)** The mRNA expression of cell cycle-promoting genes in ICP-1 treated with mimic NC + pcDNA3.1, mimic + pcDNA3.1, and mimic + pcDNA3.1-LPIN1. **(D)** The mRNA expression of preadipocyte differentiation-related genes in ICP-1 treated with mimic NC + pcDNA3.1, mimic + pcDNA3.1, and mimic + pcDNA3.1-LPIN1. **(E,F)** EdU assay performed in ICP-1 treated with mimic NC + pcDNA3.1, mimic + pcDNA3.1, and mimic + pcDNA3.1-LPIN1. **(G)** The protein expression of PPARγ and C/EBPα in ICP-1 treated with mimic NC + pcDNA3.1, mimic + pcDNA3.1 and mimic + pcDNA3.1-LPIN1. Data presented as mean ± SEM, *P < 0.05; **P < 0.01; ***P < 0.001; ****P < 0.0001; ns: no significance.

In order to further demonstrate whether miR-429-3p achieve its regulation of the biological function of preadipocytes by targeting *LPIN1*, we carried out rescue verification. We constructed the plasmid and siRNA to overexpress or knock down *LPIN1*. Results of qPCR and western blot showed that the transfections of pcDNA3.1-LPIN1 and si-LPIN1 led to overexpression and inhibition of LPIN1 mRNA and protein expression, respectively (Figures 7A,B). qPCR results showed that LPIN1 is able to fall down or reverse the promotion of miR-429-3p on regulating cell cycle-promoting genes (*Cyclin B2*, *Cyclin D1*, *Cyclin D2*, and *PCNA*) (Figure 7C). In addition, EdU assay also indicated that the expression of LPIN1 can restore the promotion of miR-429-3p on preadipocytes proliferation (Figures 7D,E). For preadipocytes differentiation, LPIN1 has also been shown to restore or reverse the function of miR-429-3p on upregulating preadipocytes diff erentiation-related genes (*PPAR*γ, *C/EBP*α, *C/EBP*β, *LPL*, and *ADIPO*) (Figure 7F). What’s more, LPIN1 overexpression reversed the PPARγ and C/EBPα protein levels upregulated by miR-429-3p (Figure 7G). These results suggested a pivotal role of LPIN1 on preadipocytes proliferation and differentiation regulated by miR-429-3p.

### *LPIN1* Suppresses Preadipocyte Proliferation

**FIGURE 8:**
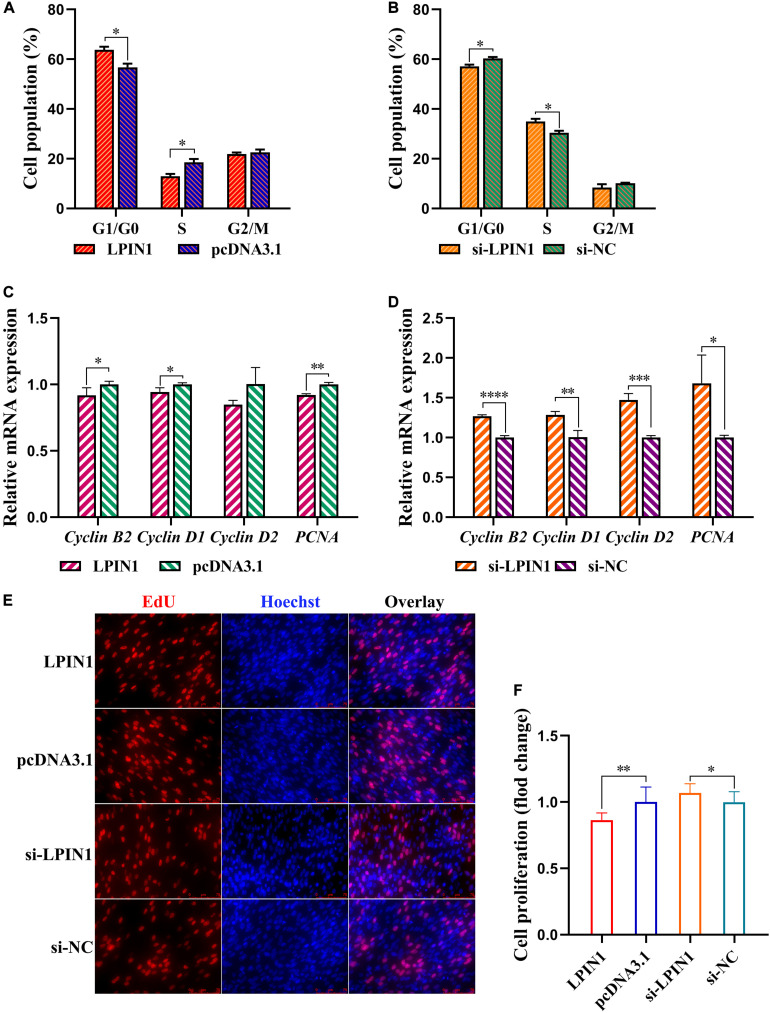
*LPIN1* suppresses preadipocytes proliferation. **(A,B)** Flow cytometry for cell cycle detection of ICP-1 cells. **(C,D)** Relative mRNA expression of *Cyclin B2*, *Cyclin D1*, *Cyclin D2*, and *PCNA* in ICP-1 cells. **(E,F)** EdU staining for cell proliferation of ICP-1 cells. Data presented as mean ± SEM, *P < 0.05; **P < 0.01; ***P < 0.001; ****P < 0.0001; ns: no significance.

Following *LPIN1* overexpression, preadipocyte cell cycle was arrested in the G1 phase, leading to a decline of the cell proportion in the S phase (Figure 8A). Conversely, *LPIN1* knockdown facilitated the preadipocyte cell cycle progression (Figure 8B). *LPIN1* overexpression upregulated the expression of *Cyclin B2*, *Cyclin D1*, *Cyclin D2*, and *PCNA* (Figures 8C,D). Results of the EdU assay showed that *LPIN1* overexpression reduced the proportion of preadipocytes in the proliferation phase, whereas *LPIN1* knockdown increased the same (Figures 8E,F). Therefore, *LPIN1* exhibited an opposite effect to that of miR-429-3p and was speculated to inhibit preadipocyte proliferation.

### *LPIN1* Inhibits Preadipocyte Differentiation by Suppressing PPARγ Pathway

**FIGURE 9:**
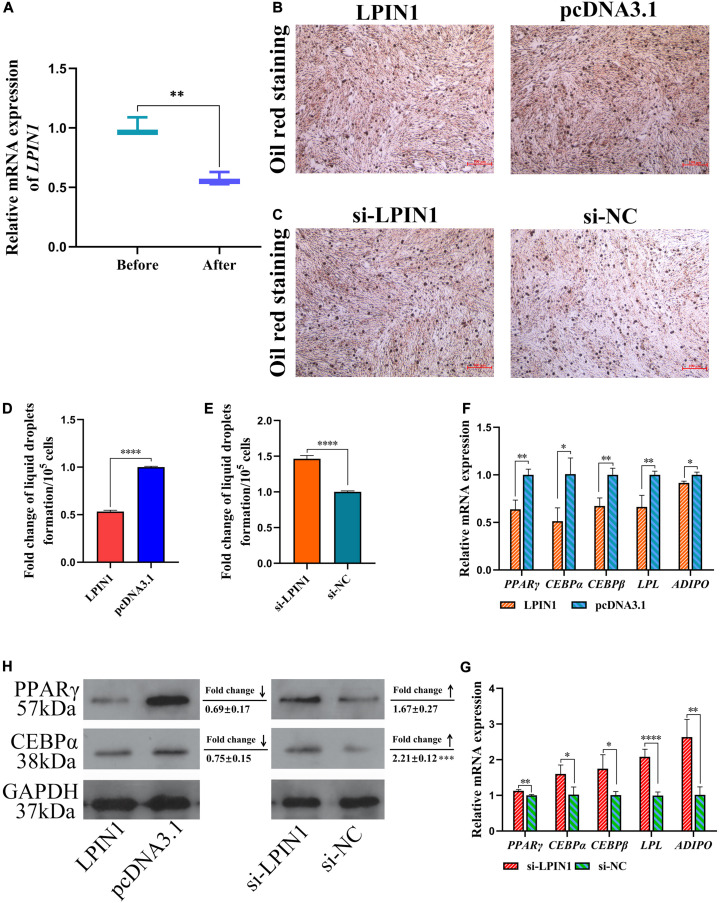
*LPIN1* suppresses preadipocytes differentiation by inhibiting PPARγ pathway. **(A)** Relative mRNA expression of *LPIN1* before and after preadipocytes differentiation. **(B,C)** Oil red staining for lipid droplet formation. **(D,E)** The fold change of lipid droplets formation per 10^5^ cells. **(F,G)** Relative mRNA expression of *PPAR*γ, *C/EBP*α, *C/EBP*β, *LP*L, and *ADIPO* in in ICP-1 cells. **(H)** The western blot for PPARγ and C/EBPα proteins in ICP-1 cells. Data presented as mean ± SEM, *P < 0.05; **P < 0.01; ***P < 0.001; ****P < 0.0001; ns: no significance.

We also determined the differential expression of *LPIN1* during adipocyte differentiation from preadipocytes. The expression of *LPIN1* was significantly lower in differentiation-induced preadipocytes than before inducing differentiation (Figure 9A). Further, Oil red O staining showed the negative effect of *LPIN1* on lipid droplet formation (Figures 9B–E). Based on the lower expression of *LPIN1* in differentiation-induced preadipocytes and abdominal fat tissues of high-fat individuals, and the relationship between *LPIN1* and miR-429-3p, we hypothesized that the low expression of *LPIN1* is responsible for PPARγ pathway activation. Moreover, qPCR results indicated that the overexpression of *LPIN1* suppressed the expression of *LPL*, *PPAR*γ, and *ADIPO*, three critical genes related to preadipocyte differentiation in PPARγ pathway, along with the expression of *C/EBP*α and *C/EBP*β (Figure 9F). On the contrary, the knockdown of *LPIN1* upregulated these genes (Figure 9G). The protein levels of CEPBα and PPARγ were reduced by overexpressing *LPIN1*, while knocking down *LPIN1* increased their protein expression (Figure 9H). Further, as shown in Figures 8F,G, *LPIN1* inhibited the formation of lipid droplets during preadipocyte differentiation. These effects of *LPIN1* are opposite to those of miR-429-3p. Collectively, these results establish that the low expression of *LPIN1* promotes the activation of PPARγ pathway and facilitates preadipocyte differentiation.

## Discussion

In chickens, abdominal fat is a major adipose tissue, and affects meat yield and quality. Recently, miRNAs have been shown to be closely involved in various biological processes including adipogenesis (Alberti et al., 2018; Guay et al., 2019; Lorente-Cebrian et al., 2019). In this study, using sequencing techniques, we identified a miRNA (miR-429-3p) and its potential target gene (*LPIN1*), which show significant relation to the process of abdominal adipose deposition. Based on our sequencing data, we designed a series of experiments at molecular and cellular levels to explore the role and the interaction of miR-429-3p and *LPIN1* in adipogenesis. Our results showed that miR-429-3p facilitated preadipocyte proliferation and differentiation. Additionally, miR-429-3p was shown to target and suppress *LPIN1*, which exerted an opposite effect to that of miR-429-3p by inhibiting preadipocyte proliferation and differentiation. Notably, miR-429-3p/*LPIN1* axis was functionally verified to modulate adipogenesis via PPARγ Pathway.

Our mRNA sequencing revealed multiple candidate genes related to lipid metabolism, including *SLC27A2*, *ACSL6*, *ABHD5*, *LPIN1*, *ADORA1*, *HNF4A*, and *SQLE*. In addition, we performed miRNA sequencing to screen some differentially expressed miRNAs which may be involved lipid metabolism, including miR-122b-3p, miR-126-5p, miR-122-5p, miR-148a-3p, miR-148b-3p, and miR-429-3p. DEMs-DEGs networks showed that miR-429-3p targeted *LPIN1* in chicken abdominal fat tissue, which indicates that miR-429-3p/*LPIN1* axis may regulate adipogenesis. miR-429-3p was significantly upregulated in high abdominal fat broilers and highly expressed after the differentiation from preadipocyte to adipocyte, indicating a potential regulatory effect on chicken adipose deposition. Given the evidence that miR-429-3p might be a significantly involved in the process of abdominal adipose deposition, we further investigated its role in this biological process. In the past decades, role of miR-429-3p has been extensively explored in tumorigenesis (Wu et al., 2018, 2019; Shen et al., 2019; Chen et al., 2020). However, little is known about its role in adipogenesis. A study by Tao et al. studied the function of miR-200b/a/429 in the regulation of metabolism in adipocytes (Tao et al., 2016). They generated adipocyte-specific miR-200b/a/429 knockout mice and found that adipocyte miR-200b/a/429 deficiency resulted in increased adiposity. Their finding suggests that miR-200b/a/429 could hinder high-fat-diet-induced obesity. However, our sequencing data showed that miR-429-3p was significantly upregulated in high-fat chickens. Flow cytometry suggested a promotion of miR-429-3p on preadipocyte cell cycle transition from G1 phase to S phase. Abdominal fat rate is influenced by abdominal fat weight and body weight, both of which are important growth traits of chickens (Ren et al., 2019; Li et al., 2020). Nucleotide polymorphisms of cell cycle related gene have been reported to be associated with chicken growth traits (Li et al., 2019), the qPCR results in our study manifested miR-429-3p indeed increased cell cycle-promoting genes expression. Taking EdU results together, we proved that miR-429-3p promoted preadipocyte proliferation. The contradictory results between the study mentioned above and our study may be attributed to the differences in animal models and experimental methods used. The animal model used in the previous study was adipocyte-specific miR-200b/a/429 knockout mice using a Cre-loxP system, in which Cre expression was driven by the aP2 promoter. In contrast, we used a chicken precursor adipocyte (ICP-1) as experiment cell model. Tao et al. analyzed the changes in body composition, metabolic parameters, energy homeostasis, glucose tolerance, and insulin sensitivity in miR-200b/a/429 knockout mice and wild-type mice after a high-fat diet, and concluded that miR-200b/a/429 knockout mice gained more body weight than the wild-type mice with increased adiposity, decreased glucose tolerance and insulin sensitivity. In another study, miR-429 has been shown to promote the proliferation of porcine preadipocyte (Peng et al., 2016), which proved by flow cytometry, qPCR and western blot of some cell cycle-related genes and EdU assay. The results of Peng et al. are consistent with ours. miR-429 also played a pivotal role on browning of white adipose tissue (Ye et al., 2020), indicating its potential role on preadipocyte differentiation. In our present study, we utilized qPCR, western blotting and oil red O staining to determine the effect of miR-429-3p on differentiation of ICP-1 through gain or loss of function experiments. We revealed the promotion effect of miR-429-3p on PPARγ signaling pathway at protein and mRNA levels. Furthermore, miR-429-3p overexpression upregulated preadipocyte differentiation-related genes expression and contributed to lipid droplets formation.

We further investigated the role of miR-429-3p in adipogenesis by focusing on the reports of an earlier study which concluded that miRNAs exert biological functions by downregulating the expression of target genes (Hausser and Zavolan, 2014). Taking DEMs-DEGs network analysis together, we predicted the potential target gene of miR-429-3p in preadipocytes and determined that *LPIN1* expression was targeted by miR-429-3p. To confirm our hypothesis, we investigated the effects of overexpression or knockdown of LPIN1 on preadipocyte proliferation and differentiation. The results demonstrated that LPIN1 exerted a completely opposite effect to that of miR-429-3p, and the mRNA and protein expression of LPIN1 was repressed by miR-429-3p. Dual-luciferase reporter assay provided the evidence of stable binding between miR-429-3p and *LPIN1* 3’UTR. A study by van Harmelen et al. (2007) showed that *LPIN1* expression was reduced in obesity, was upregulated following weight reduction in obese subjects, and was downregulated in women with the metabolic syndrome. These results are in line with our data which demonstrated a lower expression of *LPIN1* in high-fat chicken. To determine whether LPIN1 play the pivotal effect on preadipocyte proliferation and differentiation, we carried out the rescue experiments. And the result showed that the expression of *LPIN1* reversed the promotion of miR-429-3p on preadipocyte proliferation. In addition, *LPIN1* also fell back the PPARγ mRNA and protein levels, upregulated by miR-429-3p. In lipin-deficient and transgenic mouse models, lipin is identified as a key regulator in the adipocyte differentiation process in which PPARγ and C/EBPα are actively involved (Phan et al., 2004). In the chicken preadipocyte model, we also showed that LPIN1 protein is expressed during the preadipocyte differentiation process and is inversely related to PPARγ and C/EBPα expression, indicating that *LPIN1* may have inhibitory effect on chicken adipogenesis. The effect of *LPIN1* on abdominal fat deposition in chickens is completely opposite to that in mice, and the specific mechanism causing this difference is not clear, which needs further study in the future.

In conclusion, we show that miR-429-3p is upregulated, whereas *LPIN1* is downregulated in high-fat chickens. Further, we showed the relationship between miR-429-3p and *LPIN1* and established that miR-429-3p promoted preadipocyte proliferation and differentiation by inhibiting *LPIN1* activity. The findings in this study reveal a novel miR-429-3p/*LPIN1* axis involved in the progression of adipogenesis via the PPARγ pathway.

## Data Availability Statement

The datasets presented in this study can be found in online repositories. The names of the repository/repositories and accession number(s) can be found below: https://www.ncbi.nlm.nih.gov/, PRJNA657369 and https://www.ncbi.nlm.nih.gov/, PRJNA656618.

## Ethics Statement

The animal study was reviewed and approved by the Agricultural University Animal Care Committee of South China Agricultural University.

## Author Contributions

XC and LG were responsible for research designing and for most of the experiments and manuscript writing. QW participated in the part of experiments and data analysis. WH reviewed and modified the manuscript. ML, KL, and JJ participated in the data analysis and part of experiments. SL analyzed the sequencing data. QN participated in the animal experiment. WL and XZ were responsible for sample collection. QL carried out the design of the whole research and guided the research progress. All authors contributed to the article and approved the submitted version.

## Conflict of Interest

The authors declare that the research was conducted in the absence of any commercial or financial relationships that could be construed as a potential conflict of interest.

## Footnotes

1 https://dataview.ncbi.nlm.nih.gov/object/PRJNA656618

2 https://dataview.ncbi.nlm.nih.gov/object/PRJNA657369

## Supplementary Material

Supplementary Figure 1 The first nucleotide bias of small RNA.

Supplementary Table 1 Primers sequence information.

Supplementary Table 2 Data quality statistics and mapping efficiency.

Supplementary Table 3 The enriched pathways related to fat deposition.

Supplementary Table 4 The top-enrich pathways for DEGs in DAVID 6.8 platform.

Supplementary Table 5 The lipid metabolism-related pathways enriched in IPA platform.

Supplementary Table 6 miRNA count in small RNA-seq.
