# Supplementary material for: miR-429-3p/LPIN1 Axis Promotes Chicken Abdominal Fat Deposition via PPARγ Pathway
Source: Front Cell Dev Biol. 2020 Dec 21;8:595637. doi: 10.3389/fcell.2020.595637 (PMC7793751; doi:10.3389/fcell.2020.595637)
Supplement: Supplementary Figure 1 — The first nucleotide bias of small RNA. [file Data_Sheet_1.PDF]

frontiers

Supplementary Material

Supplementary Figures and Tables

Supplemental Table 1. Primers sequence information

Primers' name	Sequence (5'-3')	Product size (bp)	Annealing temperature (°C)
q-LPIN1-F	CCCAGGCAGTTGGCAAGA	200	58
q-LPIN1-R	TGGAGAATGAGTGGCCAGA		
q-Cyclin D1-F	CAGAAGTGCGAAGAGGAAGT	188	58
q-Cyclin D1-R	CTGATGGAGTTGTCGGTGTA		
q-Cyclin D2-F	AACTTGCTCTACGACGACC	150	58
q-Cyclin D2-R	TTCACAGACCTCCAACATC		
q-PCNA-F	GTGCTGGGACCTGGGTT	217	58
q-PCNA-R	CGTATCCGCATTGTCTTCT		
q-Cyclin B2-F	CAGTAAAGGCTACGAAAG	133	58
q-Cyclin B2-R	ACATCCATAGGGACAGG		
q-PPARγ-F	TCCTTCCCGCTGACCAAA	227	60
q-PPARγ-R	TCCTGCACTGCCTCCACA		
q-LPL-F	CCAAGGTAGACCAGCCATTC	154	60
q-LPL-R	TGCTCCAGGCACTTCACA		
q-ADIPOR1-F	GACAAGAACAGCAACGAGTACCGC	110	60
q-ADIPOR1-R	CCTGAAGATGCCCCGCAGAGT		

q-CEBPα-F	GACAAGAACAGCAACGAGTACCGC	195	56
q-CEBPα-R	CCTGAAGATGCCCCGCAGAGT		
q-CEBPβ-F	GCGGACTGTTTGGCTGCTCT	220	60
q-CEBPβ-R	CGGGTGAGGCTGATGTAGGTGT		
q-GAPDH-F	CAACTTTGGCATTGTGGAGG	130	56
q-GAPDH-R	CGCTGGGATGATGTTCTGG		

Supplemental Table 2. Data quality statistics and mapping efficiency.

Sample	Total Raw Reads (M)	Total Clean Reads (M)	Total Clean Bases (Gb)	Clean Reads Q30 (%)	Clean Reads Ratio (%)	Total Mapping Ratio	Uniquely Mapping Ratio
H1	21.94	21.88	1.09	88.78	99.7	90.58%	80.79%
H2	21.94	21.89	1.09	88.92	99.77	90.63%	81.21%
H3	21.94	21.88	1.09	89.2	99.71	90.25%	80.67%
H4	21.94	21.89	1.09	88.97	99.77	91.05%	81.60%
H5	21.94	21.9	1.09	89.5	99.8	90.71%	81.30%
H6	21.94	21.86	1.09	88.8	99.64	90.51%	80.93%
L1	21.94	21.86	1.09	88.62	99.62	90.11%	80.20%
L2	21.94	21.87	1.09	93.09	99.65	91.47%	82.83%
L3	21.94	21.87	1.09	89.51	99.68	90.73%	81.35%
L4	21.94	21.8	1.09	89.1	99.35	90.68%	81.31%
L5	21.94	21.89	1.09	88.98	99.74	91.32%	81.96%
L6	21.94	21.91	1.1	89.38	99.87	91.07%	81.69%

Supplemental Table 3. The enriched pathways related to fat deposition.

Pathway	Enriched genes	P value
Lipid metabolism	LPIN1, DHCR24, SQLE, HSD11B1b	0.0029
Steroid biosynthesis	DHCR24, SQLE	0.0066
Regulation of lipolysis in adipocytes	ADORA1, ABHD5	0.0377
Biosynthesis of unsaturated fatty acids	ELOVL2	1.15E-14
PPAR signaling	SCL27A2, ACSL6	1.96E-18
Fatty acid transport	FABP1	0
Peroxisome	HAO1, SCL27A2, ACSL6	1.96E-18
Lipoprotein transport	APOB	0
ECM-receptor interaction	ITGB6, SV2B, COL6A6	4.08E-5
Lipid biosynthetic process	APOA4, ELOVL2, ABHD5, CYP17A1	0

Supplemental Table 4. The top-enrich pathways for DEGs in DAVID 6.8 platform.

Pathways	DEGs
Cell adhesion molecules (CAMs)	CLDN18, PTPRF, CD8A, CD8B, CLDN3, CTLA4, CDH1, CDH3, CLDN15, ITGB8, ICOS, CNTN1, CNTNAP2, CLDN2, CD6, YF5
Glycolysis/Gluconeogenesis	GCK, ALDH1A3, ALDOB, ADH1C, ENO2, FBP1, ADH6, PCK1
Steroid hormone biosynthesis	CYP17A1, CYP2C45, CYP3A7, SD17B2, CYP7A1, HSD11B1B, UGT2A1, SRD5A2
Metabolic pathways	CYP3A7, PTGS2, ADH1C, LTC4S, PSPH, AGXT, FAH, TDO2, MAT1A, CYP7A1, DHCR24, AGPAT1, GATM, SPTLC3, HAL, FBP1, TAT, LPIN1, HAO1, NME4, CD38, CKM, DGAT2, SQLE, HSD11B1B, HAO2, UROC1, ATP6V1G3, AOC1, PRPS2, CYP2C45, ALDH18A1, HSD17B2, HSD3B7, ALDOB, HMGCS1, UPP2, ADH6, KMO, PAH, PIPOX, CMPK2, ALDH1A1, GAD2, PLCH2, ALDH1A3, PLA2G12B, PLCH1, GALNT15, ENO2, HAAO, DMGDH, UGT2A1, FUT4, PCYT1B, GCNT1, PAPSS2, GAL3ST1, ACSL6, CES1, UPB1, ACMSD, FTCD, HGD, AK7, PCK1, ACSM3, AMDHD1, CYP17A1, GCK, CYP26C1, HMGCS2, INDOL1, LIPG, ACSM5
Histidine metabolism	AMDHD1, ALDH1A3, FTCD, HAL, UROC1, AOC1
Retinol metabolism	ALDH1A1, CYP2C45, CYP3A7, CYP26C1, ADH1C, ADH6, GT2A1
Neuroactive ligand-receptor interaction	AVPR2, CCKAR, LOC770277, LHCGR, F2RL1, LPAR3, ADORA1, P2RY8, ADRB3, HTR1B, GALR2, GRPR, ADRA2C, CHRNA2, GABRG3, GABRA4, GZMA, RXFP3, NTSR1, GRM1, PLG, CHRM3, F2, P2RX2, ADRA1A, UTS2R
Calcium signaling pathway	CCKAR, TNNC2, TNNC1, ERBB3, LHCGR, MYLK2, NTSR1, GRM1, ADRB3, CD38, CAMK4, CHRM3, P2RX2, ATP2A1, GRPR, ADRA1A, CACNA1D
Cytokine-cytokine receptor interaction	CCL20, CCR10, CXCL14, CSF3, EGF, IL1R2, IL12RB1, IL20RA, IL21R, IL22RA1, IL22RA2, IL8L1, IL8L2, TNFSF13B, TNFRSF8, TNFSF15
PPAR signaling pathway	APOA2, CYP7A1, APOC3, APOA5, FABP1, FABP7, SLC27A2, ACSL6
Drug metabolism–cytochrome p450	FMO4, ALDH1A3, FMO3, ADH1C, ADH6, UGT2A1
ECM-receptor interaction	COL6A6, ITGB8, ITGB6, ITGB4, SV2B, COL2A1, VTN, LAMC2, COL11A1, THBS4
Biosynthesis of antibiotics	ALDOB, HMGCS1, FBP1, AK7, PSPH, TAT, AGXT, PCK1, NME4, HAO1, GCK, HMGCS2, SQLE, HAO2, ENO2, PAPSS2, PRPS2
Tyrosine metabolism	ALDH1A3, ADH1C, HGD, ADH6, TAT, FAH
Butanoate metabolism	ACSM3, GAD2, HMGCS2, HMGCS1, ACSM5
Biosynthesis of amino acids	ALDH18A1, MAT1A, ALDOB, ENO2, PAH, PSPH, TAT, PRPS2
Regulation of actin cytoskeleton	FGF19, FGFR2, FGF9, DIAPH3, FGF16, ITGB4, MYLK2, FGF23, PAK6, EZR,

	CHRM3, ITGB8, F2, ITGB6, PAK1, EGF
Glycerolipid metabolism	MOGAT2, DGAT2, LIPG, LPIN1, AGPAT1
Carbon metabolism	HAO1, GCK, ALDOB, HAO2, ENO2, FBP1, PSPH, AGXT, PRPS2
Metabolism of xenobiotics by cytochrome p450	ALDH1A3, HSD11B1B, ADH1C, ADH6, UGT2A1

Supplemental Table 5. The lipid metabolism-related pathways enriched in IPA platform.

Pathways	-log(p-value)	Ratio	Molecules
Insulin receptor signaling	0	0.816497	FGFR2, ASIC2, SCNN1A, PTPRE, PRK, ACA, GYS2
Fatty acid β-oxidation I	0.383	0.0625	SLC27A2, ACSL6
Phosphatidyglycerol Biosynthesis II (Non-plastidic)	0.498	0.0769	ABHD5, AGPAT1
Adipogenesis pathway	0.625	0.167	FGFR2, LPIN1, FZD10, RBP1, SOX9, KLF5, FZD5, FOXC2
cAMP-mediated signaling	0.687	0.057	HTR1B, AVPR2, ADRB3, CHRM3, CAMK4, PKIA, PDE6H, CREB3L3, ADRA2C, CAMK1G, LHCGR, PRKACA, ADORA1
Fatty acid α-oxidation	1.15	0.136	ALDH1A1, PTGS2, ALDH1A3
AMPK signaling	0.803	0.0602	FGFR2, AK9, ADRB3, AK7, ADRA2C, HNF4A, PRKACA, CHRNA2, ADRA1A
Cholesterol biosynthesis I	0.961	0.154	SQLE, DHCR24

Supplemental Table 6. miRNA count in small RNA-seq.

Sample name	Known miRNA count	Novel miRNA count
H1	488	73
H2	528	82
H3	512	95
H4	522	469
H5	495	60
H6	515	67
L1	500	165
L2	476	111
L3	494	172
L4	469	74
L5	494	89
L6	403	108
Total	886	955

H1-H6: the 6 samples in HF group; L1-L6: the 6 samples in LF group.

1.1 Supplementary Figures

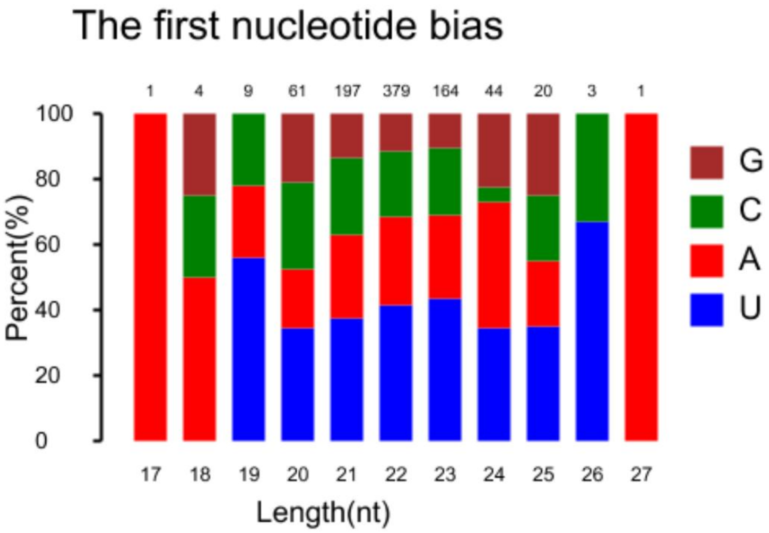

Supplemental Figure 1. The first nucleotide bias of small RNA. The X axis represents miRNAs of various lengths, and the numbers on the histogram represent the count of miRNA species of that length.
